# Evaluation of Total Phenolic Content, HPLC Analysis, and Antioxidant Potential of Three Local Varieties of Mushroom: A Comparative Study

**DOI:** 10.1155/2022/3834936

**Published:** 2022-10-19

**Authors:** Anika Tabassum Bristy, Tairin Islam, Rezwana Ahmed, Jumana Hossain, Hasan Mahmud Reza, Preeti Jain

**Affiliations:** Department of Pharmaceutical Sciences, North South University, Dhaka, Bangladesh

## Abstract

Functional foods such as mushrooms are rich in polyphenolic compounds and secondary metabolites with health-promoting properties such as antioxidant, antimicrobial, antidiabetic and immunostimulatory effects. The present study is aimed to investigate the ethanolic extracts of three varieties of mushrooms, namely, *G. lucidum*, *G. tropicum*, and *C. indica* grown in Bangladesh for phenolic and flavonoid content and their antioxidant properties. Moreover, the phenolic composition of the extracts was analyzed by using the HPLC-DAD system. *G. lucidum* extract exhibited the highest antioxidant potential as evidenced by its lowest IC_50_ value in all the tested assay models (40.44 ± 2.09 *μ*g/mL, 151.32 ± 0.35 *μ*g/mL, 137.89 ± 1.85 *μ*g/mL in DPPH, H_2_O_2,_ and NO scavenging assay, respectively) along with the highest phenolic content (81.34 ± 0.68 GAE g^−1^ extract). *G. tropicum* and *C. indica* extracts also showed significant antioxidant properties and a good amount of phenolic content, 52.16 ± 0.25 GAE g^−1^ extract, and 47.1 ± 0.26 GAE g^−1^ extract, respectively. The scavenging activity increased with the increasing concentration of extracts in all cases. The total phenolic content of the ethanolic extracts of mushroom species was highly correlated with antioxidant effects with Pearson's correlation coefficient (*r*) values ranging from 0.8883–0.9851. The *α*-amylase inhibitory and antibacterial activity of *G. lucidum* was evaluated by using 3,5-dinitrosalicylic acid and disc diffusion method, respectively. The maximum inhibitory activity recorded against *α*-amylase was 70.98 ± 0.042% at a concentration of 500 *μ*g/mL. *G. lucidum* extract exhibited the highest antibacterial activity against *Pseudomonas aeruginosa* with 23.00 ± 1.00 mm clear zone of inhibition and an MIC value of 3.5 mg/mL. The results indicate that the mushroom species tested in this study could serve as a potential source of natural antioxidants in the development of nutraceuticals and herbal drugs for the management of oxidative stress-associated diseases as well as infectious diseases.

## 1. Introduction

Reactive oxygen species (ROS) are crucial for cellular activities at physiological concentration. However, excess production of reactive oxygen species and other free radicals may lead to oxidative stress—the imbalance between the generation and accumulation of reactive oxygen species. In addition, human beings are continuously exposed to free radicals produced from chemicals, radiation, and other environmental toxins [1]. Oxidative stress is responsible for the structural modification and the disruption of physiological function of cell components which may even lead to cell death [2]. Antioxidants convert the ROS to nonreactive oxygen species, interrupt the propagation of free radicals, and inhibit their formation to break radical chain reactions for the prevention of oxidative stress-related damage [3]. Endogenous antioxidant enzymes such as glutathione peroxidase, catalase, and superoxide dismutase also deactivate free radicals and maintain normal cellular functions [4]. However, under certain conditions, the endogenous biological antioxidant defense and repair systems become inadequate to prevent oxidative damage [4, 5] and contribute to diverse diseases such as cancer, atherosclerosis, diabetes, rheumatoid arthritis, and neurodegenerative disorders like Alzheimer's and Parkinson's disease, along with early aging process [3].

The consumption of exogenous antioxidants is pivotal to support the antioxidant defense system by maintaining a sufficient level of antioxidants. Synthetic antioxidants such as butylated hydroxy anisole (BHA), butylated hydroxytoluene (BHT), and propyl gallate (PG) have been used extensively. However, over time, safety concerns have been raised due to their associated adverse effects such as skin allergies, GI issues, and cancer [6]. This has necessitated the search for effective and safe natural antioxidants which can replace synthetic ones. Numerous studies have revealed the close association between polyphenol-rich food consumption and reduced risks of heart diseases, stroke, diabetes, and cancer [7–9].

In recent times, diverse antioxidant compounds have been detected in the Fungi kingdom [10, 11]. Mushrooms are widely consumed worldwide as a functional food in fresh as well as processed forms [12]. Apart from the rich aroma, taste, and high nutritional value, some varieties of mushrooms have been reported to possess anti-inflammatory, cholesterol-lowering, hepatoprotective, antibacterial, antiviral, antidiabetic immunomodulatory, and anticancer activities [13–15]. These properties are mainly attributed to the presence of a wide range of secondary metabolites including phenolics [1, 6, 7].

Mushrooms are cultivated and consumed worldwide due to their high nutritional content and therapeutic benefits [15]. Reports indicate that crude polysaccharides of a variety of mushroom *C. indica*, commonly known as the milky mushroom, might be therapeutically effective against oxidative stress and immunodeficiency disorders [16]. *G. lucidum* or Reishi mushroom is a popular medicinal mushroom due to its health-promoting effects such as antitumor, antimicrobial, anti-inflammatory, antidiabetic, and antioxidant activities [17, 18]. *G. tropicum* is one of the major wild Ganoderma species recorded in Chinese Pharmacopeia to treat cardiovascular diseases [19]. It is known that the active constituents and therapeutic potential of plants might vary among species [20, 21] and the cultivation conditions may also contribute to such variation [21, 22]. As commercial production of mushrooms is very recent in Bangladesh, very little information is available about the nutritional benefits and therapeutic potential of the varieties of mushrooms grown in Bangladesh [15]. Against this background, the current study aimed to assess the phytochemical constituents such as phenolic and flavonoid contents and evaluate the in vitro antioxidant activity of three locally grown varieties of mushroom—*Calocybe indica*, *Ganoderma lucidum*, and *Ganoderma tropicum*. The antioxidant potential has been evaluated using multiple in vitro models combined with HPLC analysis. In addition, two in vitro assays, the *α*-amylase inhibitory activity and the antibacterial activity, were performed to assess the biological activity of *G. lucidum* extract. Results obtained from the free radical scavenging activities have been examined for their correlation with phenolic contents towards the exploration of possible uses of this plant in the development of herbal drugs and nutraceuticals.

## 2. Materials and Methods

### 2.1. Sample Collection

Three species of mushrooms used in this study were collected from the National Mushroom Development & Extension Center, Sobhanbag, Dhaka-Aricha Highway, Savar, Bangladesh. The samples were identified and authenticated by the experts in the National Mushroom Development & Extension center. The Accession no. of the three species, *C. indica*, *G. tropicum*, and *G. lucidum* are ARMT 08, ARMT 04, and ARMT 018, respectively.

### 2.2. Preparation of Extracts

The fruiting bodies of mushrooms were cleaned and washed with distilled water to remove any residual compost. These were then air-dried to constant weight and ground into a coarse powder using a laboratory scale mill and blender. Dried and powdered samples (50.0 g) were extracted with 250 mL ethanol. The extraction was carried out for 7 days accompanied by occasional shaking and stirring. The extracts were then filtered first through a piece of clean, cotton material and finally through Whatman No. 1 filter paper. The residues were extracted again with 100 mL of ethanol. The combined extracts were evaporated to dryness under reduced pressure by a rotary vacuum evaporator. The dried extracts were stored at 4°C in a refrigerator until used for analysis.

### 2.3. Preliminary Phytochemical Screening

The ethanolic extracts of *C. indica*, *G. tropicum*, and *G. lucidum* were screened for the presence of phytoconstituents such as phenolic compounds, flavonoids, tannins, saponins, and alkaloids according to standard phytochemical screening methods [23]. Wagner's reagent and Molisch's reagent were utilized to test the presence of alkaloids and carbohydrates, respectively. Flavonoids and tannins were detected with concentrated HCl and FeCl_3_, respectively [20]. Salkowski's test was used to screen for saponins and the frothing test was employed for the detection of terpenoids [20]. Unless mentioned differently in individual tests, 10% (w/v) solution of the ethanolic extract was used in each test [24].

### 2.4. Estimation of Total Phenolic Content (TPC)

Total phenolic content in the extracts was determined by the Folin-Ciocalteu assay [23]. Briefly, 0.3 mL of the extract was mixed with 1.2 mL of the Folin-Ciocalteau reagent solution (10%, v/v) and 1.5 mL of sodium carbonate solution (7.5% w/v). The absorbance was measured at 765 nm after incubation of the reaction mixture for 1 hour. A calibration curve was constructed with varying concentrations (60-150 *μ*g/mL) of gallic acid as standard. The results were expressed as mg of gallic acid equivalents (GAE) per gram extract.

### 2.5. Estimation of Total Flavonoid Content

The AlCl_3_ colorimetric method was used to estimate the total flavonoid content of the extracts [25]. Briefly, 0.5 mL of extract was mixed with 1.5 mL methanol, 0.1 mL 1 M potassium acetate solution, 2.8 mL distilled water, and 0.1 mL 10% aluminum chloride solution. To complete the reaction, the mixtures were incubated for 30 minutes at room temperature. The absorbance of the solutions was measured at 415 nm using a spectrophotometer. Total flavonoid content was calculated using quercetin standard calibration curve. Results were expressed as mg of Quercetin Equivalents (QE) per gram extract.

### 2.6. DPPH Radical Scavenging Assay

The scavenging activity of extracts was determined using DPPH free radical-scavenging assay [26]. Briefly, 0.1 mL of the extract at various concentrations was added to 2.9 mL of a 0.002% methanolic solution of DPPH. The mixture was incubated for 30 minutes in a dark place to complete the reaction at room temperature. The absorbance of the mixture at 517 nm was then measured. The capability to scavenge the DPPH radical was calculated using the following equation:

(1) $$\begin{array}{c} \text{Inhibition}\%=\left[\frac{A_{0}-A_{1}}{A_{0}}\right]\times 100. \end{array}$$

*A*_0_ is the absorbance of the control and *A*_1_ is the absorbance in the presence of the sample.

### 2.7. H_2_O_2_ Scavenging Assay

The H_2_O_2_ scavenging activity of the extracts was evaluated as described by Reddy et al. [27]. In brief, a solution of H_2_O_2_ (40 mM) was prepared in 0.1 M phosphate buffer (pH 7.4), and 3.4 mL of each extract at varying concentrations was mixed with 0.6 mL H_2_O_2_ solution. The mixtures were incubated at room temperature and the absorbance of these mixtures was recorded at 230 nm. Ascorbic acid was used as the positive control. The H_2_O_2_ scavenging activity of the extracts and standard compounds was calculated as follows:

(2) $$\begin{array}{c} \text{Scanvenging activity}\left(\%\right)=\left[\frac{A_{0}-A_{1}}{A_{0}}\right]\times 100. \end{array}$$

*A*_0_ was the absorbance of the control, and *A*_1_ was the absorbance in the presence of the sample of extract/standard.

### 2.8. Nitric Oxide Scavenging Assay

Nitric oxide scavenging activity of the extracts was estimated based on the Griess Illosvoy reaction [28]. Briefly, a reaction mixture (3 mL) containing 10 mM sodium nitroprusside and the extract solutions at varying concentrations were prepared in phosphate buffer and incubated at 25°C for 2.5 h. This was followed by the addition of 1 mL of sulfanilic acid reagent to 0.5 mL of the incubated solution and allowed to stand for 5 min. The mixture was then incubated at room temperature for 30 min after the addition of 1 mL of naphthyl ethylene diamine dihydrochloride (0.1%). Absorbance of the reaction mixtures at 540 nm was recorded. Ascorbic acid was used as a standard. The nitric oxide radical scavenging activity of the extracts was expressed as % inhibition.

(3) $$\begin{array}{c} \text{Inhibition}\%=\left(\frac{A_{0}-A_{1}}{A_{0}}\right)\text{X}100. \end{array}$$

*A*_0_ was the absorbance of the control, and *A*_1_ was the absorbance in the presence of the sample of extract/standard.

### 2.9. High Performance Liquid Chromatography (HPLC) System

HPLC-DAD analysis method was utilized to determine the phenolic composition of *C. indica*, *G. lucidum*, and *G. tropicum* extracts as described previously [29]. It was carried out on a Dionex UltiMate 3000 system equipped with a quaternary rapid separation pump (LPG-3400RS) and photodiode array detector (DAD-3000RS). Separation was performed using Acclaim® C18 (5 *μ*m) Dionex column (4.6 x 250 mm) at 30°C with a flow rate of 1 mL/min and an injection volume of 20 *μ*l. The mobile phase consisted of acetonitrile (solvent A), acetic acid solution pH 3.0 (solvent B), and methanol (solvent C) with the gradient elution program of 5%A/95%B (0-5 min), 10%A/90%B (6-9 min), 15%A/75%B/10%C (11-15 min), 20%A/65%B/15%C (16-19 min), 30%A/50%B/20%C (20-29 min), 40%A/30%B/30%C (30-35 min), and 100%A (36-40 min). The UV detector was set to 280 nm for 22.0 min, changed to 320 nm for 28.0 min, again changed to 280 nm for 35 min, and finally to 380 nm for 36 min and held for the rest of the analysis period while the diode array detector was set at an acquisition range from 200 nm to 700 nm. For the preparation of calibration curve, a standard stock solution was prepared in methanol containing arbutin (AR), (-)-epicatechin (ECA) (5 *μ*g/mL each), gallic acid (GA), hydroquinone (HQ), vanillic acid (VA), rosmarinic acid (RA), myricetin (MC) (4 *μ*g/mL each), caffeic acid (CA), Syringic acid (SA), vanillin (VL), *trans*-ferulic acid (FA) (3 *μ*g/mL each), *p*-coumaric acid (PCA), quercetin (QU), kaempferol (KF) (2 *μ*g/mL each), (+)-catechin hydrate (CH), ellagic acid (EA) (10 *μ*g/mL each), *trans*-cinnamic acid (TCA) (1 *μ*g/mL), rutin hydrate (RH) (6 *μ*g/mL), and benzoic acid (BA) (8 *μ*g/mL). A solution of the extract (10 mg/mL) was prepared in ethanol prior to HPLC analysis and all the solutions (mixed standards, sample, and spiked solutions) were filtered through 0.20 *μ*m syringe filter (Sartorius, Germany) and then degassed in an ultrasonic bath (Hwashin, Korea) for 15 min. Data acquisition, peak integration, and calibrations were calculated with Dionex Chromeleon software (Version 6.80 RS 10).

### 2.10. Alpha Amylase Enzyme Inhibition Assay

The in vitro alpha amylase inhibitory activity of *G. lucidum* extract was determined by a modified Bernfeld's method [30]. Briefly, 200 *μ*L of the test extract at concentrations ranging from 31.25-500 *μ*g/mL was mixed with 200 *μ*L of *α*-amylase enzyme solution and 200 *μ*L of 2 mM of phosphate buffer (pH −6.9). After 20 minutes of incubation at room temperature, 200 *μ*L of 1% starch solution was added to the reaction mixture and incubated further for 10 minutes at room temperature. To terminate the reaction, 200 *μ*L of 3,5-dinitrosalicylic acid (DNSA) reagent was added and the tubes are incubated for 5 minutes in a boiling water bath. The mixture was allowed to cool at room temperature and diluted with 5 mL of distilled water. The absorbance was measured by a UV-spectrophotometer at 540 nm and the *α*-amylase inhibitory activity was calculated as percent inhibition using the following formula:

(4) $$\begin{array}{c} \text{Inhibition}\left(\%\right)=\left(\frac{A_{0}-A_{1}}{A_{0}}\right)\text{X}100 \end{array}$$

*A*_0_ is the absorbance of the control, and *A*_1_ is the absorbance of the sample of extract/standard. Commercially available acarbose (Sugatrol 50 mg) was used as a standard.

### 2.11. Antibacterial Activity

#### 2.11.1. Disc Diffusion Assay

Antibacterial activity of *G lucidum* extract was determined by the disc diffusion method against five bacterial species. The strains were maintained on agar slants at 4°C and activated by subculturing at 37°C for 24 h before screening [21]. The overnight cultures grown in broth were adjusted to an inoculum size of approximately 10^6^CFU/mL following a 0.5 McFarland turbidity standard. Using a cotton swab, a suspension of the test microorganisms was swabbed on the Muller Hinton Agar (MHA) medium and the plates were allowed to dry for 5 min. For screening, sterile 6 mm diameter filter paper discs were impregnated with filter sterilized test extracts of *G. lucidum* (2 mg/disc) and placed on the surface of the seeded agar plates followed by incubation at 37°C for 24 h. Commercially available kanamycin discs (30 *μ*g/disc) were used as reference standard. Discs prepared using the solvent (DMSO) served as the negative control. Antibacterial activity was evaluated by measuring the diameters of zones of inhibition.

#### 2.11.2. Minimum Inhibitory Concentration (MIC) and Minimum Bactericidal Concentration (MBC)

The MIC of *G. lucidum* extract was determined by the broth dilution method in the Mueller Hinton broth supplemented with 10% glucose and 0.5% phenol red [22, 31]. The stock solution of the test extract was diluted to obtain concentrations from 1.0 mg/mL to 10 mg/mL. Each tube was then inoculated with an overnight culture of the appropriate bacterial strain diluted to give a final concentration of 10^6^CFU/mL. The culture tubes were incubated aerobically at 37°C for 24 h. The MIC values were taken as the lowest concentration of the extract that inhibited bacterial growth or change in color from red to yellow, resulting from the formation of acidic metabolites that corresponds to microbial growth [20, 31]. Culture media without the sample extract and the others without bacteria served as controls. For determining the MBC value, a loopful of the culture medium from the broth MIC assay sample was spread on fresh MHA plates. After incubation at 37°C for 24 h, the MBC was recorded as the lowest concentration of the test sample with no bacterial growth [22].

### 2.12. Statistical Analysis

All analyses were carried out in triplicates and the data are expressed as mean ± standard deviation (SD). GraphPad Prism software and Microsoft Excel 2007 were used for statistical analyses. One-way ANOVA and appropriate post hoc tests were performed and differences were considered significant when *p* < 0.05. For evaluation of the possible correlation between phenolic content and the free radical activities of the extracts, Pearson's correlation analysis was carried out. The IC_50_ values were determined by linear regression analysis.

## 3. Results and Discussion

Qualitative phytochemical screening of the mushroom extracts showed the presence of various secondary metabolites in ethanolic extracts (Table 1).

**Figure 1:**
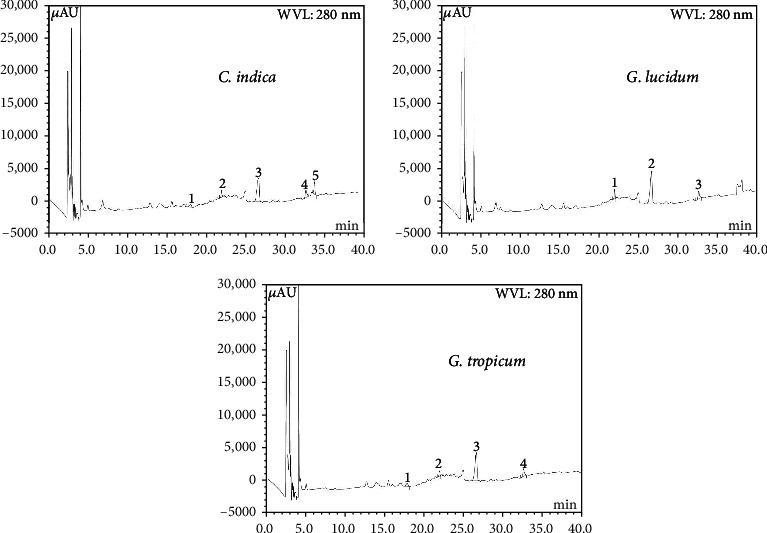
HPLC chromatogram of CI, GL, and GT extract. CI extract: Peaks: 1, (+)-catechin (CH); 2, (-)-epicatechin (ECA); 3, *trans*-ferulic acid (FA); 4, rosmarinic acid (RA). GL extract: Peaks: 1, (-)-epicatechin (ECA); 2, *trans*-ferulic acid (FA); 3, rosmarinic acid (RA). GT extract: Peaks: 1, (+)-catechin (CH); 2, (-)-epicatechin (ECA); 3, *trans*-ferulic acid (FA); 4, rosmarinic acid (RA).

Total phenolic and flavonoid contents in the mushroom extracts, expressed as mg GAEs/g of extract and mg QEs/g of extract, respectively, are shown in Table 2. The total phenolic content (TPC) in *G. lucidum* extract (81.34 ± 0.68 mg GAE/g) was significantly higher than that in *C. indica* (52.16 ± 0.03 mg GAE/g) and *G. tropicum* (47.13 ± 0.23 mg GAE/g). TPC found in the ethanolic extract of locally grown *G. lucidum* was higher than that reported by Sheikh et al. (71.43 ± 0.94 mg GAE/g) [32] and by Celik et al. (49.52 ± 0.68 mg GAE/g) [33]. This suggests that *G. lucidum* could be used as a potential source of natural antioxidants. Total flavonoid content in the ethanolic extract of *G. tropicum* (29.31 ± 0.38 mg QE/g) was slightly higher than that in *C. indica* and *G. lucidum* (Table 2). Polyphenolics are one of the major contributors to the antioxidant properties of mushrooms [7–12]. HPLC-DAD system was used for the identification and quantification of polyphenols in the ethanolic extracts of the examined mushrooms species. The chromatographic separations of polyphenols in the tested extracts are shown in Figure 1. The content of each compound was calculated from the corresponding calibration curve and presented as the mean of five determinations as shown in Table 3. Compounds such as catechin, epicatechin, *trans*-ferulic acid, rosmarinic acid, and myricetin were detected from the fruiting bodies of *C. indica* (Table 3; Figure 1). High amount of myricetin (11.47 mg/100 g extract), a natural flavanol of significant pharmacological importance, was observed in *C. indica* extract. Antioxidant and free radical scavenging activities have been widely studied by researchers and interestingly, myricetin was reported to be more effective than *α*-tocopherol as an antioxidant in liposomes [34, 35]. *Trans*-ferulic acid (TFA) content was highest in *G. lucidum* extract (11.93 mg/100 g extract), followed by *C. indica* (8.85 mg/100 g extract) and *G. tropicum* (8.09 mg/100 g extract) extracts. TFA is known to exhibit anti-inflammatory, cytoprotective, and antioxidant effects and also prevents lipid peroxidation in biological systems [36]. Catechin was found in the highest amount in *G. tropicum* extract (10.53 mg/100 g extract) whereas epicatechin was highest in *G. lucidum* extract (8.67 mg/100 g extract). Due to their potent antioxidant properties, catechins are found to be beneficial in preventing and protecting from pathologies caused by oxidative stress [37]. These results demonstrate that the tested mushroom species contain several important polyphenols, which possess important pharmacological effects. Hence, these locally grown varieties of mushrooms could find potential use in the development of herbal medicines, nutraceuticals, and natural preservatives.

Investigation of antioxidant activity through multiple methods accounts for various mechanisms for antioxidant action, and thereby provides a strong assessment of the antioxidant potential of any sample. Antioxidants differ in their chemical nature as well as scavenging mechanisms such as elimination of peroxide, free radical-mediated chain termination, and hydrogen donation [38]. Hence, the use of multiple methods is required for evaluation of the antioxidant potential of plant extracts. In this study, three complimentary test systems, DPPH radical scavenging assay, H_2_O_2_ scavenging assay, and nitric oxide scavenging assay, have been utilized to assess the antioxidant capacity of ethanolic extracts of *C. indica*, *G. lucidum*, and *G. tropicum*. The scavenging potential of the extracts was expressed in terms of IC_50_ values (Table 4). A stronger ability to scavenge the free radical is indicated by a lower IC_50_ value.

**Figure 2:**
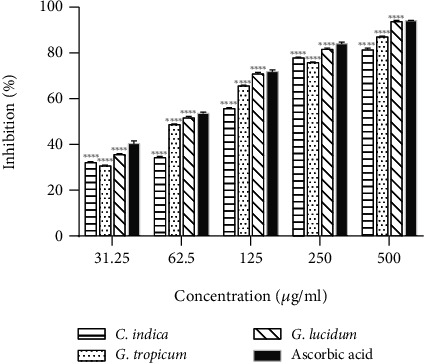
DPPH radicals scavenging effects of various ethanolic mushroom extracts in comparison with ascorbic acid. Results are expressed as mean ± SD (*n* = 3). ^∗∗∗∗^*p* < 0.0001 vs. standard (ascorbic acid) at each concentration.

DPPH scavenging assay is based on the ability of DPPH to react with proton donors. DPPH, the stable free radical, possesses a characteristic absorption at 517 nm which decreases when it reacts with the antioxidants. This decrease in absorption is determined as a measure of the extent of free radical scavenging capability. The use of DPPH radical is advantageous as this free radical is unaffected by side reactions including enzyme inhibition [39].  Figure 2 shows the concentration-dependent DPPH scavenging activity of the mushroom extracts (*R*^2^ = 0.7836 − 0.8086). The scavenging activity of the extracts followed the sequence *G. lucidum* > *G. tropicum* > *C. indica*. At a concentration of 500 *μ*g/mL, the scavenging ability of the *G. lucidum* extract is 93.78 ± 0.42%, whereas the value was 81.43 ± 0.67% for *C. indica* and 87.01 ± 0.30% for *G. tropicum.* The lowest activity was observed in *G. tropicum* having 30.76 ± 0.24% scavenging at the concentration of 31.25 *μ*g/mL. *G. lucidum* extract also showed lowest IC_50_ value (40.44 ± 2.1 *μ*g/mL) among the mushroom extracts tested (Table 4), indicating the strongest scavenging potential. Standard compound ascorbic acid showed IC_50_ value of 10.78 ± 0.65 *μ*g/mL indicating stronger scavenging activity as compared to the mushroom extracts (*p* < 0.05). Varied scavenging effectiveness of *G. lucidum* extracts has been reported previously. Sheikh et al. from India reported DPPH scavenging efficiency for ethanolic extract of *G. lucidum* with IC_50_ of 13.615 *μ*g/mL [32] while the methanolic extract from *G. lucidum* [40] was reported to have 142.84 ± 71.84 *μ*g/mL. Celik et al. [33] from Turkey reported an IC_50_ value of 7.03 ± 0.07 *μ*g/mL with ethanolic extract of *G. lucidum*. Such variations in scavenging activity could be attributed to geographical origin, environmental growth conditions and the type of solvents used for extraction.

**Figure 3:**
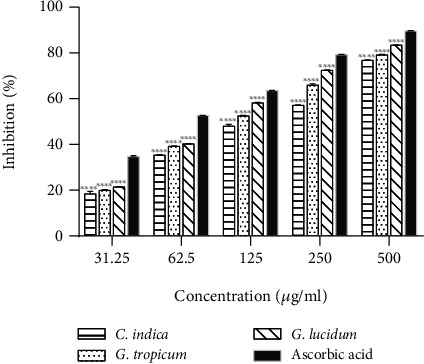
H_2_O_2_ radicals scavenging effects of various ethanolic mushroom extracts in comparison with ascorbic acid. Results are expressed as mean ± SD (*n* = 3). ^∗∗∗∗^*p* < 0.0001, ^∗∗^*p* < 0.01 vs. standard (ascorbic acid) at each concentration.

All the mushroom extracts were efficient in scavenging hydrogen peroxide (Figure 3) in a dose-dependent manner (*R*^2^ = 0.7953 − 0.8975). As shown in Figure 3, *G. lucidum* showed the highest free radical inhibition at all the tested concentrations whereas *C. indica* extract exhibited the lowest efficiency. Hydrogen peroxide results in tissue damage in living systems [21] due to its rapid conversion into hydroxyl free radicals (OH) [24]. The ability of mushroom extracts to scavenge H_2_O_2_ indicates their cytoprotective activity, which could be of potential use in the development of herbal medicines. IC_50_ value of *G. lucidum* extract (151.32 ± 0.35 *μ*g/mL, Table 4) obtained in this study was significantly lower than a previously reported value of 822.188 *μ*g/mL by Radhika et al. from India [41] for ethanol extract of *G. lucidum*. The strong hydrogen peroxide scavenging efficiency of *G. lucidum* as demonstrated in the present study could be attributed to its highest total phenolic content (Table 2).

**Figure 4:**
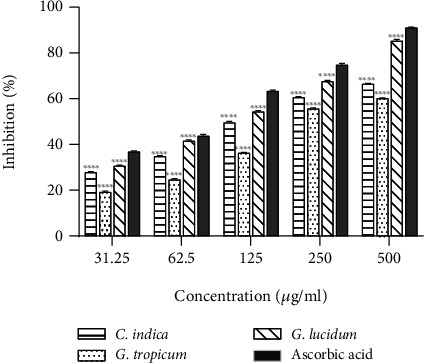
NO radicals scavenging effects of various ethanolic mushroom extracts in comparison with ascorbic acid. Results are expressed as mean ± SD (*n* = 3). ^∗∗∗∗^*p* < 0.0001 vs. standard (ascorbic acid).

Nitric oxide, an unstable free radical, is known to induce various inflammatory responses leading to tissue toxicity, carcinoma, and pathological conditions such as multiple sclerosis, arthritis, and ulcerative colitis. Nitric oxide reacts with oxygen and generates stable nitrate and nitrites in cells. The free radical scavengers compete with nitric oxide for oxygen and inhibit the overproduction of nitrite ions which mitigates the propagation of inflammation [42]. All the mushroom extracts tested in this study efficiently scavenged nitric oxide radical in a dose-dependent manner (*R*^2^ = 0.8168 − 0.9346). The highest NO scavenging activity was exhibited by *G. lucidum* extract (Figure 4) with IC_50_ value of 137.89 ± 1.85 *μ*g/mL (Table 4), which is significantly lower than that reported by Radhika et al. from India (860.184 *μ*g/mL) [41] for NO scavenging by ethanolic extract of *G. lucidum*. The results demonstrate that *G. lucidum* possesses strong NO scavenging property. In this study, *G. lucidum* extract scavenged 52% nitric oxide radical at 100 *μ*g/mL, which is lower than that reported by Sheikh et al. (63%) [32] from India at the same concentration. These variations in scavenging efficiency in different studies may be attributed to the difference in growth conditions, source, handling, and time of extraction.

**Figure 5:**
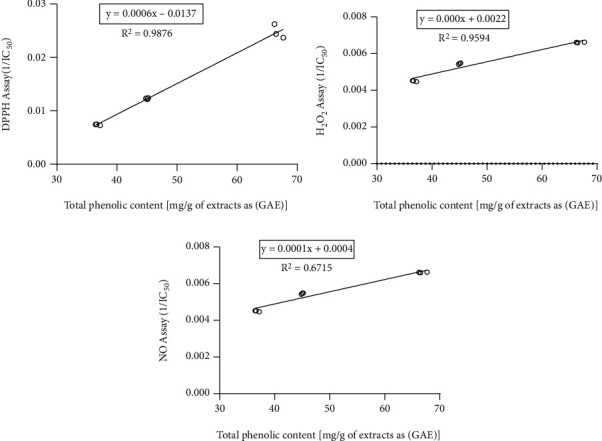
Correlation between total phenolic content and (a) DPPH radical scavenging activity of the extracts. Correlation coefficient, *r* = 0.9851 and coefficient of determination, *R*^2^ = 0.9705. (b) H_2_O_2_ radical scavenging activity of the extracts. Correlation coefficient, *r* = 0.9449 and coefficient of determination, *R*^2^ = 0.8928. (c) NO radical scavenging activity of the extracts. Correlation coefficient, *r* = 0.8883 and coefficient of determination, *R*^2^ = 0.7890.

Total phenols are considered as the major natural antioxidant components and contributors to the biological activities of medicinal plants including mushrooms [20, 32, 41]. In this study, the correlation between total phenolic content in the mushroom extracts and antioxidant activity was evaluated. Pearson's correlation coefficient (*r*) and coefficient of determination (*R*^2^) were determined for each assay model. As shown in Figure 5, a positive correlation was observed between total phenolic content (TPC) and 1/IC_50_ values of extracts in antioxidant assays with the Pearson value (*r*) ranging from 0.8883 to 0.9851. Strong positive correlation was found between TPC and DPPH scavenging (*r* = 0.9851, *R*^2^ = 0.9705) and H_2_O_2_ scavenging (*r* = 0.9449, *R*^2^ = 0.8928) activity. Relatively lower correlation was observed with NO scavenging (*r* = 0.8883, *R*^2^ = 0.7890) activity.

**Figure 6:**
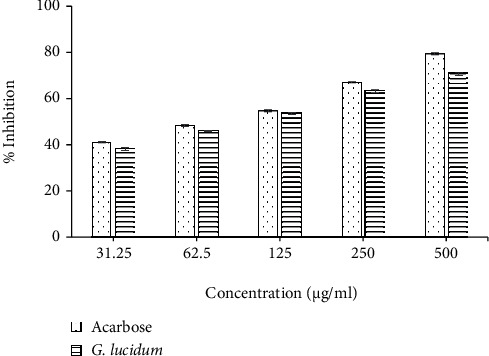
*α*-Amylase inhibitory activity of ethanolic extract of *G. lucidum* in comparison with the standard (Acarbose). Results are expressed as mean ± SD (*n* = 3).

The results obtained in this study are in line with published studies that report correlation between the antioxidant activities and total phenolics, thereby suggesting phenolics as the major contributor towards the antioxidant activity [32, 43, 44] in mushrooms. *G. lucidum* extract demonstrated the highest scavenging activity compared to other extracts in all assay models used in this study, which could be attributed to its high total phenolic content. In order to further explore the medicinal effects of *G. lucidum* extract, two in vitro biological assays were performed, namely, the *α*-amylase inhibitory activity and antibacterial activity. Use of antioxidants has been found effective in reducing the severity of complications linked with oxidative stress-associated diseases such as diabetes [45]. *G. lucidum* supplementation has exhibited reduced fasting blood sugar, glycosylated hemoglobin, and improved insulin resistance in animal models [46]. Previously, some studies have reported aldose reductase, *α*-glucosidase, and *α*-amylase inhibitory activities of *G. lucidum* [18, 40, 46–48]. The *α*-amylase enzyme is normally found in the pancreatic juice and saliva which helps to break down starch into absorbable molecules. Inhibitors of this enzyme delay the breakdown of carbohydrates in the small intestine and help to control the postprandial blood glucose elevation [49], hence are valuable to diabetic patients. In this study, ethanolic extract of *G. lucidum* showed 70.98 ± 0.042% of *α*-amylase inhibition (Figure 6) at the highest test concentration (500 *μ*g/mL). The extract exhibited a dose-dependent increase in the percentage of enzyme inhibition. The standard, acarbose, showed an IC_50_ value of 88.05 ± 2.96 *μ*g/mL, whereas the *G. lucidum* extract inhibited the enzyme with an IC_50_ value of 124.41 ± 3.40 *μ*g/mL. This value found for *G. lucidum* is lower than that reported by Uddin et al. [50] for methanolic mushroom extract (386.04 *μ*g/mL) indicating stronger *α*-amylase inhibitory activity of the ethanolic extract. The results obtained from *α*-amylase inhibition assay suggest that *G. lucidum* can be used as a functional food for diabetics and also serve as a natural source of antidiabetic drugs.

The antibacterial activity of *G. lucidum* extract was evaluated against five bacterial strains (Table 5) by disc diffusion method. Maximum antibacterial activity was recorded against *Pseudomonas aeruginosa* and *Escherichia coli* showing clear zones of inhibition of 23.00 ± 1.00 and 22.67 ± 1.15 mm, respectively. The effect was similar to the standard drug kanamycin against *Pseudomonas aeruginosa* (Table 5), a gram-negative bacterium considered to be responsible for infections in the lung, skin, eye, and urinary tract [20]. This suggests that *G. lucidum* may find applications in treating infections caused by *Pseudomonas aeruginosa*. Significant activities were also recorded against *Salmonella typhi* (16.67 ± 1.15 mm) and *Shigella flexneri* (16.33 ± 1.15 mm), and the results were comparable with kanamycin (Table 5). However, no antibacterial effect was observed against *Staphylococcus aureus* in this study. The standard drug, kanamycin showed zones of inhibition ranging from 18.33 ± 0.58 to 27.00 ± 1.00 mm against all the test organisms. The negative control, solvent DMSO, showed no inhibitory effect on the organisms used in this study. Our results are in line with the previous studies [51, 52] that reported the antibacterial property of *G. lucidum*. In this study, ethanol extract of locally grown *G. lucidum* showed higher activity against bacteria *S. aureus*, *E. coli*, *S. typhi*, and *P. aeruginosa* compared to that in a study by Quereshi et. al, [51] which indicates the local variety of *G. lucidum* could serve as a potential source of antimicrobial agents. The significant antibacterial effect exhibited by *G. lucidum* extract could be attributed to the presence of polyphenolics and compounds such as alkaloids, tannins, and saponins as revealed through phytochemical screening. MIC and MBC values were determined against organisms for which antimicrobial activity was observed in the zone inhibition assay. *G. lucidum* extract exhibited the lowest MIC and MBC values of 3.5 and 4.5 mg/mL, respectively, against both *E. coli.* and *P. aeruginosa*. MIC and MBC values of the extract were found to be 4.0 and 5.5 mg/mL, respectively, against *S. typhi* while against *S. flexneri*, MIC and MBC values obtained were 4.5 and 6.5 mg/mL, respectively. Varying MIC values ranging between 0.20 mg/ml -8.0 mg/ml for different solvent extracts of *G. lucidum* have been reported previously [52, 53] against various organisms. A study on methanolic extracts of 3 edible mushrooms (*Pleurotus ostreatus*, *Lentinula edodes*, and *Hypsizigus tessulatus*) cultivated in Bangladesh reported MIC values ranging from 1 mg/mL to 9 mg/mL against test organisms [54]. Variation in source, species, and the choice of extracting solvent account for the differences in pharmacological activities [22]. Hence, further studies using different solvent extracts and a wider range of test organisms could provide more insights into the antibacterial potential of locally grown *G. lucidum*.

## 4. Conclusion

The present study revealed that *Calocybe indica*, *Ganoderma lucidum*, and *Ganoderma tropicum* possess significant antioxidant and free radical scavenging activities which could be correlated to their phenolic content. Among the three local varieties of mushroom tested, *G. lucidum* exhibited the highest antioxidant potential and also demonstrated significant *α*-amylase inhibitory and antibacterial activities. This suggests that locally grown varieties of mushrooms especially, *G. lucidum* could serve as a potential source of natural antioxidants and antibacterial agents. Inclusion of these mushrooms in diet and as a functional food could help in alleviating pathologies linked with oxidative stress-associated diseases such as diabetes, degenerative disorders, and aberrant immune response. The significant antibacterial activity demonstrated by *G. lucidum* extracts against gram-negative bacteria used in this study indicates that it can be exploited as a natural drug in the treatment of several infectious diseases. However, isolation and identification of the specific compounds responsible for the biological activities and in vivo studies are needed for better clinical applications.

## Figures and Tables

**Table 1:** Phytochemical analysis of the ethanolic extracts of the three mushroom species.

Species	Flavonoid	Carbohydrate	Phenol	Tannin	Steroid	Saponin	Terpenoid	Alkaloid
*C. indica*	**++**	**—**	**++**	**++**	**+**	**++**	**+**	**—**
*G. tropicum*	**++**	**+**	**++**	**+**	**+**	**—**	**+**	**—**
*G. lucidum*	**++**	**+**	**+++**	**++**	**+**	**+**	**—**	**+**

‘++' denotes present in high concentration; ‘+' denotes present in small concentration; ‘-' denotes absent.

**Table 2:** Total phenolic and flavonoid content in ethanolic extracts of the three mushroom species.

Species	Total phenolic content Mg GAE/g extract	Total flavonoid content Mg QEE/g extract
*C. indica*	47.13 ± 0.23^a^	29.17 ± 0.3^a^
*G. tropicum*	52.16 ± 0.03^b^	29.31 ± 0.38^b^
*G. lucidum*	81.34 ± 0.68^c^	29.2 ± 0.55^c^

Results are expressed as mean ± SD (*n* = 3). Values in the same column followed by a different letter superscript (a-c) are significantly different (*p* < 0.05).

**Table 3:** Contents of polyphenolic compounds in the three mushroom extracts (*n* = 5).

Polyphenolic Compound	*C. indica* extract		*G. lucidum* extract		*G. tropicum* extract	
	Content (mg/100 g of dry extract)	% RSD	Content (mg/100 g of dry extract)	% RSD	Content (mg/100 g of dry extract)	% RSD
CH	9.03	0.07			10.53	0.08
ECA	7.61	0.05	8.67	0.06	5.66	0.03
FA	8.85	0.07	11.93	0.13	8.09	0.05
RA	2.72	0.02	2.75	0.02	2.61	0.01
MC	11.47	0.10				

CH (catechin), ECA (Epicatechin), FA (*trans-*ferulic acid), RA (rosmarinic acid), MC (myricetin).

**Table 4:** IC_50_ (*μ*g/mL) values of various ethanolic mushroom extracts and standard in different antioxidant assays.

Extracts	DPPH	H_2_O_2_	NO
*C. indica*	135.17 ± 1.67^a^	221.96 ± 1.42^a^	219.96 ± 1.41^a^
*G. tropicum*	81.34 ± 0.79^b^	183.27 ± 1.25^b^	315.74 ± 2.27^b^
*G. lucidum*	40.44 ± 2.1^c^	151.32 ± 0.35^c^	137.89 ± 1.85^c^
Standard^∗^	10.78 ± 0.65^d^	61.01 ± 1.59^d^	83.58 ± 1.51^d^

^∗^Positive reference standard (ascorbic acid). Results are expressed as mean ± SD (*n* = 3).

Values in the same column with different letter superscript letter (a-c) indicate that they are significantly different (*p* < 0.05).

**Table 5:** Antibacterial activity of *G. lucidum* extract.

Tested bacteria	Zone of inhibition (mm)	
	*G. lucidum* extract	Standard (kanamycin)
		
*Staphylococcus aureus* (ATCC 25923)	00	22.33 ± 0.58
*Escherichia coli* (ATCC 25922)	22.67 ± 1.15	27.00 ± 1.00
*Pseudomonas aeruginosa* (ATCC 27853)	23.00 ± 1.00	23.33 ± 0.57
*Salmonella typhi* (ATCC 6539)	16.67 ± 1.15	20.67 ± 0.58
*Shigella flexneri* (ATCC 12022)	16.33 ± 1.15	18.33 ± 0.58

## Data Availability

The data used to support the findings of this study are available from the corresponding author upon request.
